# Retrospective stable isotopes of vertebrae reveal sexual ontogenetic patterns and trophic ecology in oceanic whitetip shark, *Carcharhinus longimanus*

**DOI:** 10.1002/ece3.8452

**Published:** 2021-12-23

**Authors:** Yongfu Shen, Yi Gong, Feng Wu, Yunkai Li

**Affiliations:** ^1^ College of Marine Sciences Shanghai Ocean University Shanghai China; ^2^ Laboratory for Marine Fisheries Science and Food Production Processes Qingdao National Laboratory for Marine Science and Technology Qingdao China; ^3^ The Key Laboratory of Sustainable Exploitation of Oceanic Fisheries Resources Ministry of Education Shanghai China; ^4^ National Engineering Research Centre for Oceanic Fisheries Shanghai Ocean University Shanghai China

**Keywords:** *Carcharhinus longimanus*, ontogeny, stable isotopes, vertebra

## Abstract

There is a common phenomenon in nature whereby some animals have differences in their ontogenetic changes in dietary preferences between sexes, especially apex predators. These reflect changes in the needs of development during their lifetimes. Apex predators potentially have diverse dietary niches and a large impact on the trophic dynamics within ecosystems. However, the difference in life history between males and females often leads to increased difficulty in management and conservation. In this study, 25 oceanic whitetip sharks, *Carcharhinus longimanus*, were collected from the central and eastern tropical Pacific. Retrospective stable isotope analysis of vertebrae was used to evaluate the potential ontogenetic differences in feeding habits and niche width between sexes. Results showed that *C. longimanus* had a wide range of δ^13^C values (−18.1 to −12.3‰) and δ^15^N values (8.9–14.8‰). However, males and females had similar trophic positions with large niche overlap at similar growth stages. Both sexes had increasing δ^13^C values but relatively constant δ^15^N values along the vertebrae. These results indicated that male and female *C. longimanus* may share similar feeding strategies and movement patterns. The results presented in this study enhance our understanding of sexual ontogenetic patterns and ecological role of *C. longimanus* and highlighted the applicability of vertebrae for characterizing shark life‐history traits.

## Abstract 2

In this study, 25 oceanic whitetip sharks, *Carcharhinus longimanus*, were collected from the Central and Eastern Pacific. Retrospective stable isotope analysis of vertebrae was used to evaluate the feeding habits and niche breadth differences between the sexes. The results showed that the oceanic whitetip shark is a long‐lived oceanic pelagic shark with generalist feeding behavior.

## 1 INTRODUCTION

Most oceanic pelagic sharks are highly migratory predators playing complex but critical roles in marine ecosystems (Bonfil et al., 2008). The oceanic whitetip shark, *Carcharhinus longimanus*, is an apex marine predator potentially completing its entire life cycle in the open ocean (Backus et al., 1956; Bonfil et al., 2008; Mather & Day, 1954). Characteristics associated with high longevity, late maturity, slow growth rate, and low fecundity make this once abundant species experience severe population declines throughout its global range due to overfishing (D'Alberto et al., 2017; Myers et al., 2007; Ward & Myers, 2005). The decline of large predatory species was reported to reduce the natural mortality in a range of their preys and trigger trophic cascade changes in many marine ecosystems (Ferretti et al., 2010). Recently, the oceanic whitetip shark was listed as Critically Endangered by the International Union for the Conservation of Nature (IUCN), as well as being classified to Appendix II of the Convention on International Trade in Endangered Species (CITES) in 2013 (Rigby et al., 2019).

The feeding habits of large predatory sharks usually change through ontogeny, primarily due to morphological changes accompanying growth, age‐specific habitat use, foraging tactics, and reproductive requirements (Estupiñán‐Montaño et al., 2018; Heupel et al., 2007; Werner & Hall, 1976). Spatial and sexual segregations were observed in several oceanic shark species, which makes their stock assessment and conservation more complicated (Tsai et al., 2015). Although previous observations speculated on the opportunistic feeding behavior of the *C*. *longimanus*, knowledge of its foraging ecology is still fragmentary, especially its ontogeny and sexual variation due to its highly migratory behavior and the inaccessibility of oceanic habitat (Baum & Worm, 2009; Hussey et al., 2010; Newman et al., 2012). Such information is crucial for their conservation and efficient fishery management but also to preserve pelagic ecosystem functioning since sharks are often the keystone species in marine food webs (Grubbs, 2010; Madigan et al., 2021).

Stable carbon and nitrogen isotope ratios (δ^13^C and δ^15^N, respectively) in metabolically inert tissue, such as shark vertebrae, are efficient intrinsic markers for elucidating ontogeny/sexual variations in diet and/or habitat use of many shark species, such as the white shark *Carcharodon carcharias* (Estrada et al., 2006; Kerr et al., 2006; Kim et al., 2012; Wolf et al., 2009), blue shark *Prionace glauca* (Curnick et al., 2019; Estupiñán‐Montaño et al., 2019), salmon shark *Lamna ditropis* (Carlisle et al., 2014), and three hammerhead shark species *Sphyrna mokarran*, *S*. *lewini*, and *S*. *zygaena* (Loor‐Andrade et al., 2015; Raoult et al., 2019). This approach is based on the fact that shark vertebrae are related to lifetime information deposited in the growth bands reflecting the entire life histories of individuals (Estrada et al., 2006).

In this study, the isotopic time series from the successive vertebral sections of *C*. *longimanus* were investigated to evaluate the ontogeny and sexual variations in diet and trophic positions of this ecologically important species with the aim of getting a better understanding of the ecological role of *C*. *longimanus* in the central and eastern tropical Pacific.

## 2 MATERIALS AND METHODS

### 2.1 Sample collection

**FIGURE 1:**
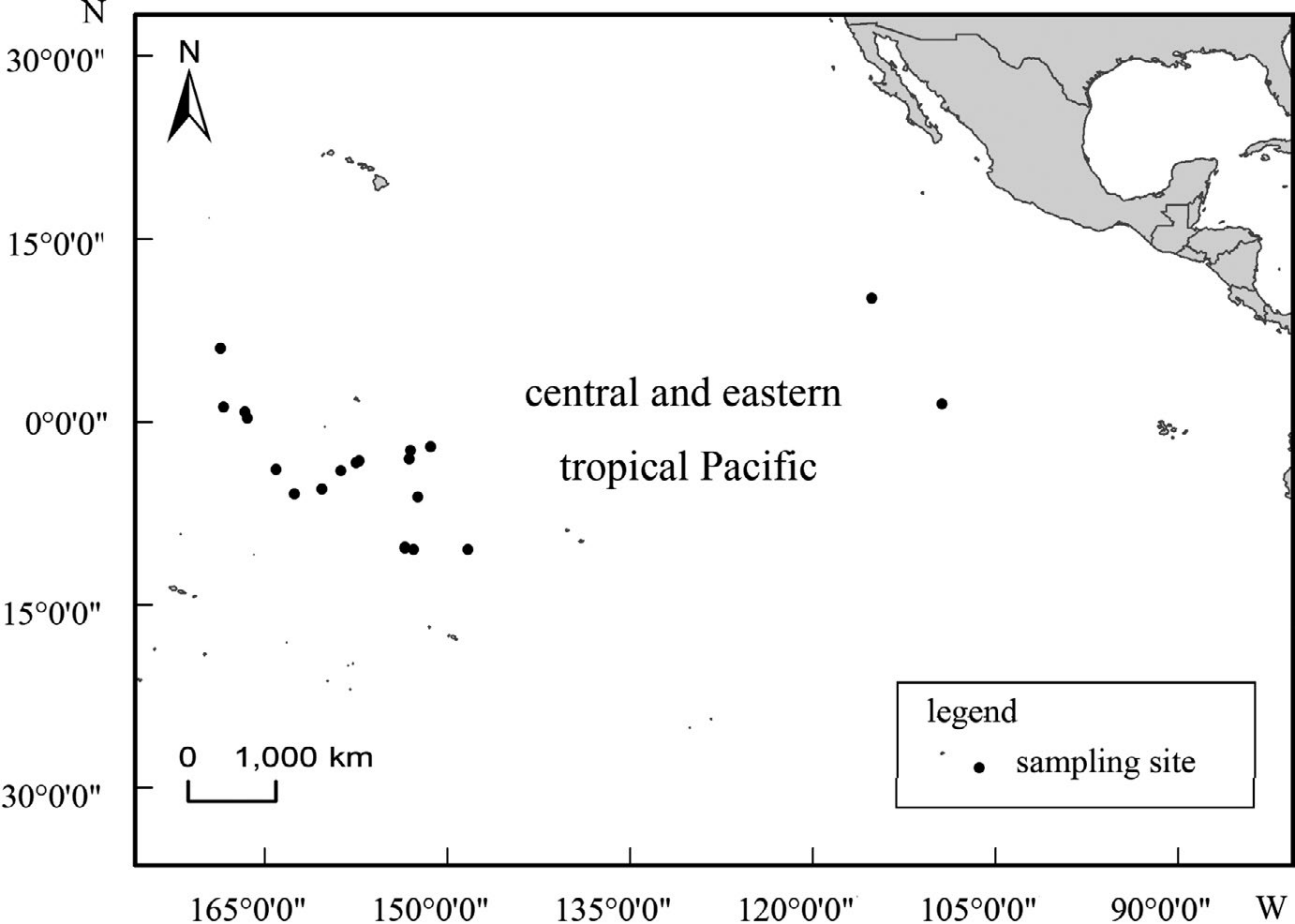
Sampling locations in the central and eastern tropical Pacific

A total of 25 *C*. *longimanus* specimens were obtained from the bycatch of Chinese tuna longline vessels operating in the central and eastern tropical Pacific from 2010 to 2019 (Figure 1). The total length and sex of each individual were recorded (Table 1). Vertebrae columns were taken from the dorsal‐anterior part of the shark body, between the head and first dorsal fin, cleaned using ultrapure water, and stored at −40°C until being transported to the laboratory.

**TABLE 1:** Biological data of *Carcharhinus longimanus* in the central and eastern tropical Pacific

Sample number	Total length/cm	Age	Sex	Maturity
OCS‐1‐1	191.8	5	Male	Mature
OCS‐1‐9	190.0	5	Female	Immature
OCS‐4‐2	158.9	5	Male	Immature
OCS‐4‐4	179.5	7	Male	Mature
OCS‐4‐7	185.0	9	Female	Mature
OCS‐4‐9	176.7	7	Male	Immature
OCS‐5‐1	175.4	6	Female	Immature
OCS‐5‐4	189.1	7	Male	Mature
OCS‐6‐1	168.0	6	Male	Immature
OCS‐6‐4	172.6	11	Female	Immature
OCS‐6‐5	230.2	14	Male	Immature
OCS‐6‐8	164.4	7	Male	Immature
OCS‐6‐10	235.0	16	Female	Mature
OCS‐6‐11	227.4	14	Male	Mature
OCS‐9‐1	205.5	12	Male	Mature
OCS‐9‐3	167.1	8	Male	Immature
OCS‐9‐4	170.0	7	Male	Immature
OCS‐10‐1	242.0	13	Female	Mature
OCS‐2‐10	171.3	9	Female	Immature
OCS‐8‐1	165.6	10	Female	Mature
OCS‐8‐2	150.0	9	Female	Immature
OCS‐8‐3	142.8	6	Female	Immature
OCS‐8‐10	153.6	9	Male	Immature
OCS‐9‐10	165.0	8	Male	Immature
OCS‐34‐8	160.3	4	Female	Immature

### 2.2 Preparation for age reading

**FIGURE 2:**
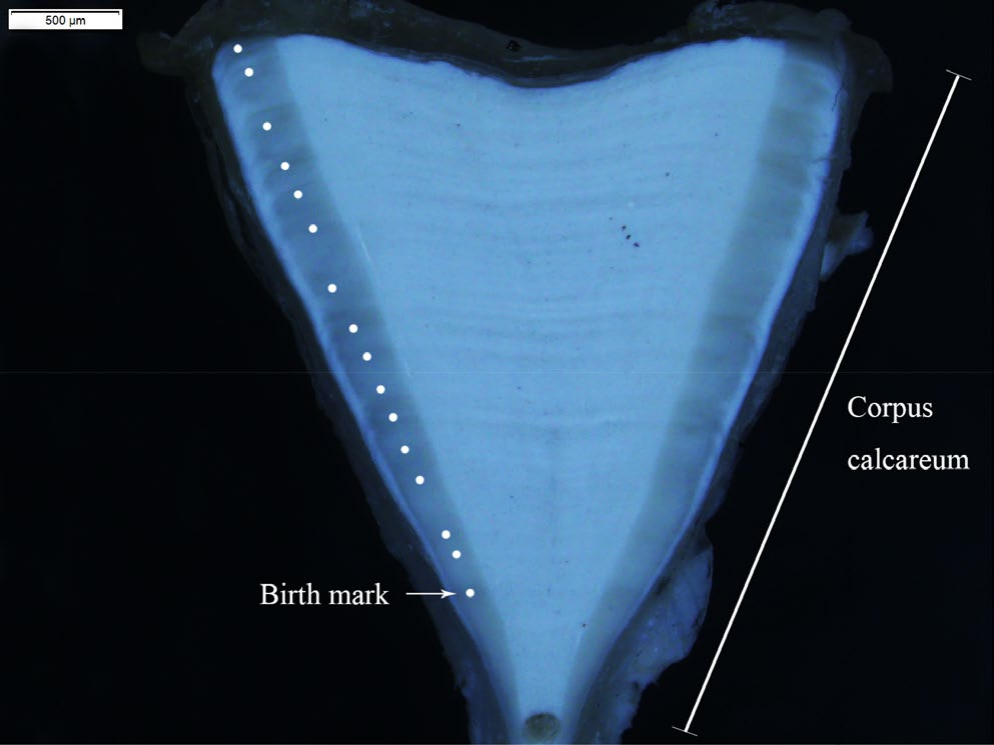
Photograph of a vertebral section of a female *Carcharhinus longimanus*, estimated to be 16 years old, at 235 cm total length

The neural arch and connective tissue were removed from each vertebra. Then, the vertebrae were dried in drying ovens at 60°C. To distinguish the growth bands, we polished the vertebrae with 120, 600, and 1200 μm grit in sequence to optimize the visualization of growth bands on the sagittal plane. After the age estimation by two readers, the vertebrae were sampled from the birth ring toward the outside edge to obtain vertebral collagen samples, using a micro‐drill with a 0.5 mm drill bit (Figure 2).

### 2.3 Stable isotope analysis (SIA)

Due to the high density of the edge, we sampled every two‐growth band after the eighth annulus band. To remove residual inorganic carbon, powdered vertebral samples were placed in 1.5 ml of ethylenediaminetetraacetic acid (EDTA) solution at 0.5 M for a week. EDTA was preferred over hydrochloric acid (HCl) because it was less likely to dissolve the sample. Once the process was complete, the samples were rinsed five times with deionized water and placed into a drying oven at 60°C for 24 h. Approximately 0.2–2 mg of samples were weighed into tin capsules and analyzed using an IsoPrime 100 isotope ratio mass spectrometer (IsoPrime Corporation, Cheadle, UK) and a vario IsoPrime cube elemental analyzer (Elementar Analysensysteme GmbH, Hanau, Germany).

The C/N ratio can be estimated to determine whether the treatment applied to the vertebral collagen was effective. A ratio ≤3.5 indicates that demineralization has been effective (Hussey et al., 2012). The isotope compositions of the samples are expressed in δ^13^C and δ^15^N notation and were calculated using the following equations:

$$\delta^{13}C\left(‱\right)=\left(\frac{{\left({}^{13}C{/}^{12}C\right)}_{\text{sample}}}{{\left({}^{13}C{/}^{12}C\right)}_{\text{standard}}}‐1\right)\times 1000$$

$$\delta^{15}N\left(‱\right)=\left(\frac{{\left({}^{15}N{/}^{14}N\right)}_{\text{sample}}}{{\left({}^{15}N{/}^{14}N\right)}_{\text{standard}}}‐1\right)\times 1000$$

where ‰ is parts per thousand; ^13^C/^12^C and ^15^N/^14^N are the atomic ratios of ^13^C and ^15^N in the sample or standard, respectively; δ is the heavy‐to‐light isotope ratio in the sample. The standard reference materials for C and N were Pee Dee Belemnite carbonate and air, respectively. The reference standards USGS 24 (−16.049‰ vPDB) and USGS 26 (53.7‰ vN_2_) were used for the quantification of the ^13^C and ^15^N stable isotope values, respectively. Every tenth sample was run in triplicate with a laboratory reference standard (Protein (−26.98‰ vPDB and 5.96‰ vN_2_)) to assess the within‐run precision, and a blank sample was run every ten samples to remove residual gases. The analytical errors of the δ^13^C and δ^15^N values were approximately 0.13‰ and 0.06‰, respectively.

### 2.4 Statistical analysis

Due to male sharks matured at 8.8 ± 1.2 years, and the females matured at 8.6 ± 1.2 years (Shen et al., 2021), the sharks were grouped into eight categories: those from 1–7 years old were divided into seven groups, and those older than 8 years were classified as adult individuals. The ontogenetic isotopic enrichment patterns were inferred from a sampling starting point located at the birth ring (Figure 2). The relative enrichment of ^13^C and ^15^N was calculated using the following equations by Estrada et al. (2006):

$$\text{Enrichment}\;Y=\frac{\delta^{z}Y_{n}‐\delta^{z}Y_{\text{birth}}}{\delta^{z}Y_{n}}$$

where *Y* is the element of interest (^13^C or ^15^N), *z* is the atomic mass of the element, and *n* is the number of growth bands. The relative trophic position (TP) was estimated using the following equation:

$$\text{TP}=\frac{\log\left(\delta^{15}N_{\text{lim}}‐\delta^{15}N_{\text{base}}\right)‐\log\left(\delta^{15}N_{\text{lim}}‐\delta^{15}N_{\text{TL}}\right)}{k}+{\text{TP}}_{\text{base}}$$

where TP_base_ is the trophic position of baseline species, δ^15^N_lim_ is the saturating isotope value, *k* represents the rate at which δ^15^N_TP_ approaches δ^15^N_lim_, and δ^15^N_TP_ is the δ^15^N value of the shark. The signatures of the zooplankton (δ^13^C = −20.1 ± 0.7‰ SD and δ^15^N = 5.3 ± 0.8‰ SD; Estupiñán‐Montaño et al., 2019) sampled in the study area were used as an isotopic baseline with TP_base_ = 2. The δ^15^N_lim_ and *k* values of 21.93 and 0.14, respectively, were derived from a meta‐analysis of experimental isotope data (Hussey et al., 2014).

The niche width and isotopic overlap between individuals, sexes, and growth stages were estimated using the Stable Isotope Bayesian Ellipses method in R, with analysis using ellipses, calculated by a covariance matrix that defines their shapes and areas (Jackson et al., 2011), to the Bayesian estimate of the standard ellipse area (SEA_B_). Isotopic overlap between sexes at each growth stage was then inferred using a Bayesian approach implemented in the R package “nicheROVER.” Overlap estimates were generated from 1000 posterior draws based on 95% probabilistic niche regions (Swanson et al., 2015).

Statistical analyses were performed using SPSS 22.0. All the stable isotope data were tested for normality using the Shapiro–Wilk test (*p *> .05). Parametric (ANOVA, and paired *t*‐test) or non‐parametric (Kolmogorov–Smirnov test and Wilcoxon signed‐rank test) analyses of variance were used to test for isotopic differences between categories (sex and growth stages). Ontogenetic variations were analyzed by linear regression. Post hoc multiple comparison tests (Tukey's test and Dunn's test) were then performed to identify specific differences between categories. All results are presented as the mean ± SE.

## 3 RESULTS

A total of 181 samples of vertebral collagen were obtained from 25 individuals (14 males, 101 vertebral sections; 11 females, 80 vertebral sections). After removing residual inorganic carbon, the range of the C/N ratio was 2.7 ~ 3.5, indicating that the demineralization was sufficient (Table 2).

**TABLE 2:** δ^13^C and δ^15^N as a function of age (in years), maturity stage, and trophic level for *Carcharhinus longimanus* in the central and eastern tropical Pacific

Maturity stage	Sex	Age	δ^13^C (‰)		δ^15^N(‰)		Trophic position	
			Range	Mean ± SE	Range	Mean ± SE	Range	Mean ± SE
Immature	Male	1	−17.6 to −12.8	−14.1 ± 0.3	9.8–13.4	12.2 ± 0.3	3.0–4.1	3.8 ± 0.1
	Female		−17.0 to −12.8	−14.6 ± 0.4	9.5–14.2	11.7 ± 0.5	2.9–4.4	3.4 ± 0.2
	Combined		−17.6 to −12.8	−14.7 ± 0.3	9.5–14.2	12.2 ± 0.3	2.9–4.4	3.7 ± 0.1
Immature	Male	2	−17.1 to −13.1	−14.3 ± 0.2	10.2–14.8	12.8 ± 0.3	3.1–4.6	3.9 ± 0.1
	Female		−17.3 to −12.9	−14.7 ± 0.3	9.2–14.2	11.7 ± 0.4	2.8–4.4	3.5 ± 0.1
	Combined		−17.3 to −12.9	−14.6 ± 0.2	9.2–14.8	12.3 ± 0.3	2.8–4.6	3.7 ± 0.1
Immature	Male	3	−18.1 to −13.2	−14.4 ± 0.3	9.0–14.0	11.9 ± 0.4	2.8–4.3	3.6 ± 0.1
	Female		−17.4 to −13.8	−15.1 ± 0.4	8.9–13.2	11.7 ± 0.4	2.8–4.0	3.6 ± 0.1
	Combined		−18.1 to −13.2	−14.9 ± 0.2	8.9–14.0	11.8 ± 0.3	2.8–4.3	3.6 ± 0.1
Immature	Male	4	−17.1 to −12.8	−14.3 ± 0.3	10.1–14.3	12.4 ± 0.4	3.1–4.4	3.7 ± 0.1
	Female		−17.6 to −13.2	−14.9 ± 0.4	9.5–13.7	11.1 ± 0.4	2.9–4.2	3.2 ± 0.1
	Combined		−17.6 to −12.8	−14.4 ± 0.3	9.5–14.3	11.7 ± 0.3	2.9–4.4	3.4 ± 0.1
Immature	Male	5	−16.3 to −12.5	−13.9 ± 0.2	10.9–14.3	12.5 ± 0.3	3.3–4.4	3.7 ± 0.1
	Female		−17.8 to −13.2	14.5 ± 0.3	9.4–12.9	11.3 ± 0.4	2.9–3.9	3.3 ± 0.1
	Combined		−17.8 to −12.5	−14.5 ± 0.4	9.4–14.3	11.6 ± 0.3	2.8–4.4	3.6 ± 0.1
Immature	Male	6	−16.5 to −12.6	−14.0 ± 0.3	10.2–14.4	12.2 ± 0.4	3.1–4.5	3.7 ± 0.1
	Female		−16.1 to −13.2	−14.3 ± 0.3	9.4–12.6	11.2 ± 0.4	2.9–3.8	3.5 ± 0.1
	Combined		−16.5 to −12.6	−14.0 ± 0.2	9.4–14.4	11.9 ± 0.3	2.9–4.5	3.5 ± 0.1
Immature	Male	7	−15.8 to −12.3	−13.7 ± 0.4	9.9–14.5	12.2 ± 0.5	3.0–4.5	3.7 ± 0.1
	Female		−18.1 to−12.7	−14.4 ± 0.7	9.4–13.2	12.1 ± 0.5	2.9–4.0	3.5 ± 0.1
	Combined		−18.1 to −12.3	−14.2 ± 0.4	9.4–14.5	12.2 ± 0.3	2.9–4.5	3.7 ± 0.1
Adults	Male	≥8	−16.2 to −12.4	−12.9 ± 0.2	10.5–13.9	12.9 ± 0.4	3.2–4.3	4.0 ± 0.1
	Female		−16.2 to −13.0	−13.7 ± 0.3	10.0–13.4	11.9 ± 0.3	3.0–4.1	3.6 ± 0.1
	Combined		−16.2 to −12.1	−14.2 ± 0.3	10.0–13.9	12.2 ± 0.3	3.0–4.3	3.7 ± 0.1
Overall	Male	–	−18.1 to −12.3	−14.3 ± 0.1	9.0–14.8	12.3 ± 0.1	2.8–4.6	3.7 ± 0.1
	Female		−18.1 to −12.7	−14.7 ± 0.2	8.9–14.2	11.5 ± 0.1	2.8–4.4	3.5 ± 0.1
	Combined		−18.1 to −12.3	−14.5 ± 0.1	8.9–14.8	12.0 ± 0.1	2.8–4.6	3.7 ± 0.1

### 3.1 Stable isotope values and trophic position

The δ^13^C range for all *C*. *longimanus* specimens was −18.1 to −12.3‰ (−14.5 ± 0.1‰). Males had a wider range of δ^13^C values than females (δ^13^C _males_: −14.3 ± 0.1‰, ranged from −18.1 to −12.3‰; δ^13^C_females_: −14.7 ± 0.2‰, ranged from −18.1 to −12.7‰). No difference in δ^13^C values (combining males and females) was observed among growth stages (ANOVA, *p* = .303). There was no difference across growth stages either for male or females (Tukey's test, *P*_male_ = 0.682, and *P*_female_ = 0.431, respectively). However, most females exhibited lower δ^13^C values than males in each growth stage indicating potential sexual variation (paired *t*‐test, *p* = .040) (Table 1).

The δ^15^N range for all *C*. *longimanus* was 8.9–14.8‰ (12.0 ± 0.1‰). The δ^15^N values varied between 9.0‰ and 14.8‰ (12.3 ± 0.1‰) for the males and 8.9‰ and 14.2‰ (11.5 ± 0.1‰) for the females. There was no difference in δ^15^N values among growth stages (for males and females combined: ANOVA, *p* = .063, for male and female separately: Tukey's test, *P*_male_ = 0.696 and *P*_female_ = 0.832, between sexes: paired *t*‐test, *p* = .695).

The mean trophic position of *C*. *longimanus* was estimated to be 3.7 ± 0.1 with a range of 2.8–4.7 (Table 2). The TP estimated by age suggested similar TP (*p* > .05) throughout ontogeny. And the estimated TP for males and females was also similar at each growth stage. The estimated TPs for the growth stages of males (3.7 ± 0.1) and females (3.5 ± 0.1) showed no significant differences (*p* > .05).

### 3.2 Ontogenetic variations in isotopic values

**FIGURE 3:**
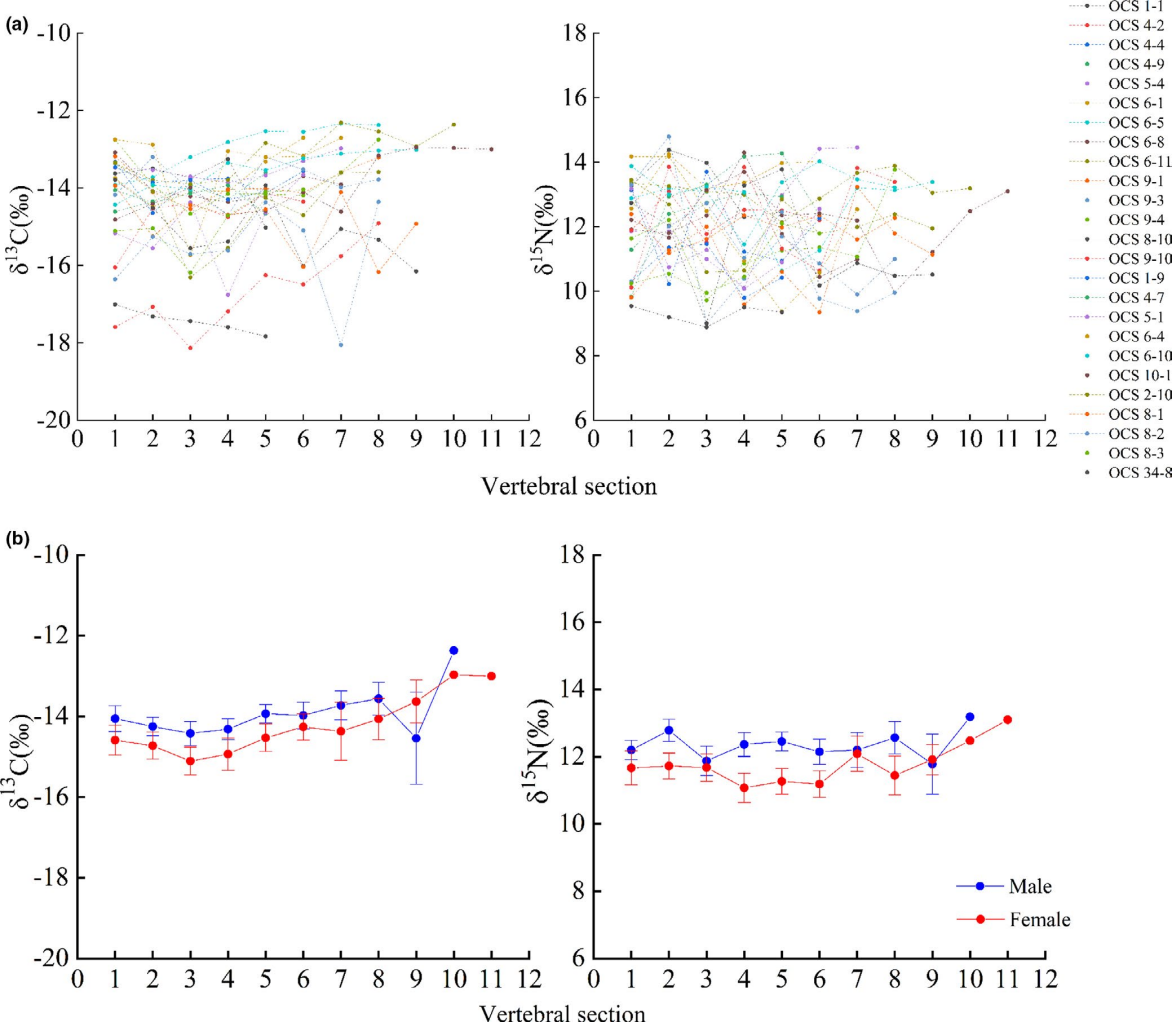
δ^13^C and δ^15^N values obtained from vertebrae of *Carcharhinus longimanus* sampled (*n* = 25). (a) Individual patterns, (b) Mean ± SE

Except for a few individuals (5/25, 20%), most *C*. *longimanus* had increasing but variable δ^13^C values along the vertebrae. Meanwhile, δ^15^N values were more variable, since only seven individuals showed linear relationships with age. For all male or female individuals, the linear regression models showed general trends of slowly increasing δ^13^C values along the vertebral sections, but not for the δ^15^N values (Figure 3). Specifically, after 3 years old, the δ^13^C values increased slowly for both males and females with the highest values observed in the last section (after 11 years old). The δ^15^N values of both sexes slightly fluctuated all the time, and the values at maturity were generally increasing.

**FIGURE 4:**
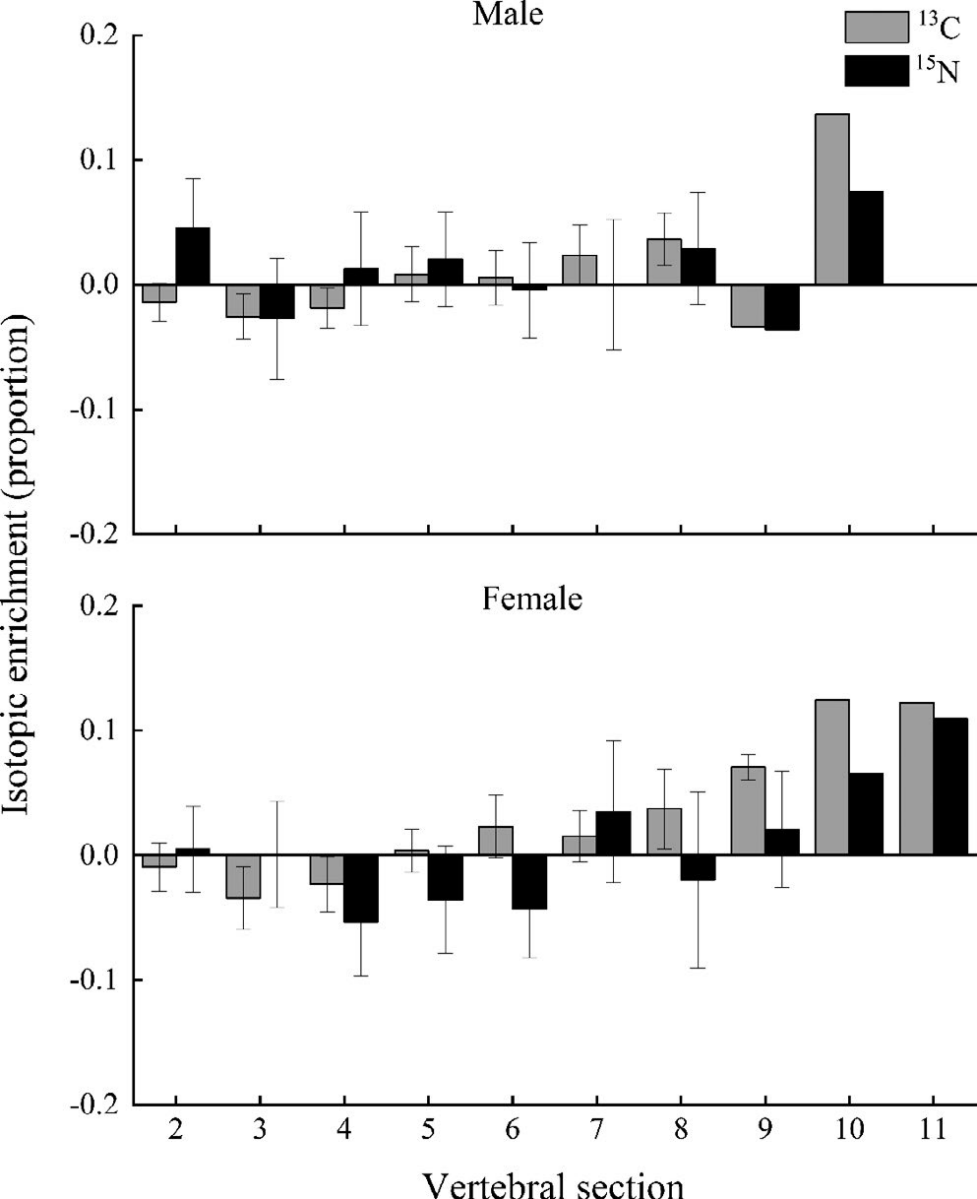
Isotopic enrichment (mean ± SE) of ^13^C (gray) and ^15^N (black) in the *Carcharhinus longimanus* vs. annulus bands, relative to values between the birth band and the first band sampling location

The reconstructions of the ^15^N isotopic enrichment patterns of the 25 individuals showed differences in characteristics between sexes, but similar characteristics for the ^13^C isotopic enrichment patterns for both males and females. The vertebral collagen samples of both sexes taken before the fourth sections of the vertebrae were depleted in ^13^C, relative to the sampling starting point located at the first section. By contrast, samples taken at other sections from the centrum of the vertebrae were enriched in ^13^C overall (Figure 4). A clear ^15^N depletion was also observed in the life history of immature females from the first section of the vertebrae, except for the second section for females, which showed little enrichment in ^15^N (Figure 4). Males showed enrichment for most of their life histories, except for the third, sixth, and ninth sections, which showed slight ^15^N depletion.

### 3.3 Isotopic niche

The overall estimated SEA_B_ was 6.01‰^2^, suggesting that *C*. *longimanus* has a broad isotopic niche. A broad isotopic niche was estimated for both sexes (for the male, SEA_B_ was 5.89‰^2^, and for the female, SEA_B_ was 5.62‰^2^). In every growth stage, the niche width of males was higher than that of females, except for 1‐ and 4‐year‐olds (Table 3). The largest niche width was exhibited by adult males.

**TABLE 3:** Niche width and isotopic overlap of *Carcharhinus longimanus* by maturity stage (separated sexes)

Maturity stage	Age	Isotopic niche (SEA_B_ [‰^2^])		Overlap size (‰^2^)	Isotopic niche overlap (SEA_B_ [%])	
		Male	Female		Male	Female
Immature	1	3.90	5.89	3.36	86.15	57.05
Immature	2	4.88	4.42	2.77	56.76	62.67
Immature	3	7.24	4.74	4.55	62.85	95.99
Immature	4	6.53	6.56	5.91	90.51	90.09
Immature	5	4.90	2.65	1.95	39.80	73.58
Immature	6	3.88	3.05	1.97	50.77	64.59
Immature	7	5.64	4.77	2.98	52.84	62.47
Mature	≥8	7.05	4.07	3.97	56.31	97.54

**FIGURE 5:**
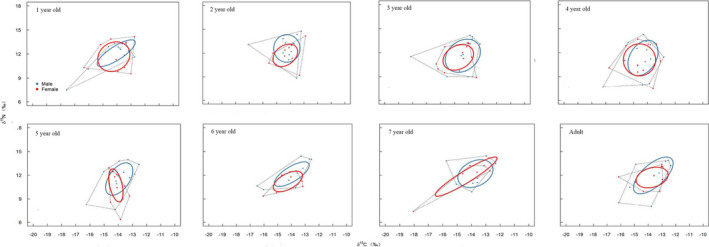
The niche overlap between sexes and among growth stages of *Carcharhinus longimanus* in the central and eastern tropical Pacific. The ellipses represent the estimated standard ellipse area determined by the SIBER analysis

For both sexes, large niche overlaps were observed between growth stages (36.79%~99.99%). The niche overlap between sexes was also high (66.01%). But the overlap of isotopic niches between males and females exhibited no linear relationships with age (*R* ^2^ = .02, *p *> .05), which was mainly driven by females shifting isotopic niches (i.e., decreasing δ^13^C isotope values at 5 years old) through time, while males remained at relatively similar δ^13^C values. Essentially, based on the niche width of the males, the niche overlap between sexes was high at all growth stages, while for females, the niche overlap was only higher at 1 and 4 years old (Table 3, Figure 5).

## 4 DISCUSSION

This study was the first attempt to use the stable isotope ratios of vertebrae to reveal the long‐term trophic patterns of *C*. *longimanus* and improved our understanding of its dietary ontogeny, trophic positions, and isotopic niche width.

### 4.1 Habitat use and trophic position

Variance in isotopic compositions from vertebrae reflects the integration of metabolism, growth, protein composition, and feeding (Estupiñán‐Montaño et al., 2019). Feeding is the key determinant of δ^13^C and δ^15^N values in the tissue, which are influenced by the exogenous supply of isotopes (MacNeil et al., 2005). The wide range of δ^13^C values of all specimens (~5.8‰) suggested intraspecific variation in dietary habits and sources. Generally, ^12^C of aqueous CO_2_ is preferentially uptake by phytoplankton during photosynthesis, resulting in the enrichment of ^13^C. Higher δ^13^C values, therefore, can be observed in waters with high phytoplankton productivity or warmer water closer to the equator (Graham et al., 2010). Major marine and marginal marine habitat types had distinct δ^13^C values and could provide different basic carbon sources. Such variation can be transferred along the food chain and reflected in the tissues of top predators (Hobson et al., 1994). It was reported that the *C*. *longimanus* typically lives offshore, along the edges of continental shelves or around oceanic islands in water deeper than 184 m, and from the surface to at least 152 m depth (Backus et al., 1956; Bonfil et al., 2008). And *C*. *longimanus* was considered to be an oceanic species and was able to undertake long‐distance migration and vertical movement throughout its entire lifetime (Madigan et al., 2015). It was also possible that this species regularly migrated to breeding grounds as a foraging strategy. Although the species spent most of its time in less than 200‐m depth, it could conduct deep dives into the mesopelagic zone for a short period, appearing to tolerate colder waters (Howey et al., 2016; Tolotti et al., 2017). Meanwhile, small *C*. *longimanus* have been observed to inhabit deep reef areas along the continental shelf where upwelling processes lead to enhanced productivity (i.e., high δ^13^C values) (Seki et al., 1998). In addition, Howey‐Jordan et al. (2013) reported that *C*. *longimanus* depart from habitats associated with reproduction or take roundtrip between favorable expansion of warm water and more northerly latitude areas. However, there was currently no definitive evidence of parturition or mating occurred (e.g., the presence of neonates or juveniles, or observations of sharks with physical signs of mating) in the Pacific (Madigan et al., 2015). Such complex movement patterns might contribute to the wide range of δ^13^C values. Differences observed in δ^13^C values between males and females at each growth stage may indicate the sexual dimorphism and site segregation (Canani & Oddone, 2020). However, isotopic discrimination at higher trophic positions also could be driven by physiological processes (Madigan et al., 2021). This might be also the reason for the difference between sexes in δ^13^C, but further investigation is needed.

In this study, we found a broad range of δ^15^N values in *C*. *longimanus*, possibly driven by maternal transfer or ontogenetic changes in diets (Kiszka et al., 2014). The consumption of prey at different trophic positions could be reflected in the vertebrae. In addition, the wide range of δ^15^N values could also be attributed to environmental factors (Lin, 2013). The upwelling of Equatorial waters that undergoes a reduction in NO^3−^ generates residual nitrates enriched in ^15^N, leading to primary production with high δ^15^N values, which would result in apparent jumps of one or two units in the trophic chain (Estupiñán‐Montaño et al., 2019). Thus, offshore waters typically have lower δ^15^N values compared with nearshore though vertical layers may also exhibit different δ^15^N values (Palacios, 2002). Typically, opportunistic predators with long‐distance feeding migration behaviors have wide ranges of δ^15^N values (Howey et al., 2016; Madigan et al., 2015; Young & Carlson, 2020). Moreover, individual physiological and biochemical factors might also contribute to the isotopic variation (Shipley & Matich, 2020). Boggs et al. (2016) have confirmed nutritional condition and reproduction could affect isotopic fractionation. Both the males and females showed similar δ^15^N values at each growth stage. This pattern indicated similar feeding strategies in different habitats, supported by the differences in the δ^13^C values between sexes at each growth stage.

The estimated TP of *C*. *longimanus* is 3.7 ± 0.1 across all growth stages. It was slightly lower than the previous studies using stomach content analysis (TP_mean_ = 4.2; Cortés, 1999), but was similar with the bulk stable isotope analysis using white muscles (TP_mean_ = 3.92 ± 0.25; Li et al., 2014). It was worth noting that unidentified turnover rates and the enrichment mechanism of stable isotopes in vertebrae could affect the result of isotopic values and further investigation is required. Although there was a wide range across the studied period, the males and females in this study had similar TP among all growth stages.

### 4.2 Dietary ontogeny

Several studies had reported ontogenetic changes in the habitat or diet of pelagic sharks (Kim et al., 2012; Li et al., 2016; Young et al., 2010). Such patterns also occurred in our study with a high degree of individual specialization within the population. The δ^13^C values of both sexes increased with growth but lack of the corresponding trends in δ^15^N values, implying that feeding cannot explain the δ^13^C change. Therefore, the increases in δ^13^C values versus ages reflected the ontogenetic changes in habitat use, such as the long‐distance and vertical movement by their migratory behavior, complementing their diet, giving birth, and exploring new ecosystems (Howey‐Jordan et al., 2013; Young & Carlson, 2020). In addition, maternal transfer might also affect δ^13^C values in early life. As aplacental viviparous sharks, female *C*. *longimanus* transferred nutrients directly to their offspring through the yolk sac placenta. Vaudo et al. (2010) reported depleted δ^13^C values of embryos of the blacktip shark, *Carcharhinus limbatus*, compared with their mothers. *C*. *longimanus* might have a similar transfer pattern resulting in higher δ^13^C values in adults than those of juveniles. Similar trends of δ^13^C values in this species were also found in Atlantic (Madigan et al., 2015).

Though variable shifts in δ^15^N values along the vertebrae for both sexes were observed, no discernible trend was detected. Essentially, the δ^15^N values of 1‐year‐old were similar to the values of adult. This might reflect the maternal isotopic signature acquired through maternal transfer (McMeans et al., 2009). Moreover, higher δ^15^N values were found in 7‐ or 8‐year‐old, which might be explained by a diet toward large size fish and squid (Cortés, 1999). This hypothesis was supported by our isotopic enrichment analysis in which the enrichment in ^15^N was observed in adults, especially in the females. The result of isotopic enrichment analysis suggested that compared with females, male sharks have lower energy requirement change with growth (Papastamatiou et al., 2018). In addition, the enrichment of δ^15^N values also could be affected by environmental factors (Lin, 2013). Oceanic sharks are believed highly migratory, undertake ontogenetic migration among different habitats within 1 year (Howey‐Jordan et al., 2013). Alternatively, in the oligotrophic open ocean, sharks exhibit opportunistic feeding in their life span. Thus, due to these dilution effects, the dietary ontogeny of *C*. *longimanus* may be even more pronounced than the results suggest (Madigan et al., 2015).

The isotopic enrichment of 25 individuals found in this study suggested that the maternal δ^13^C signatures were “erased” by the offspring at 5 years old, and the maternal δ^15^N values were “erased” at 5 years old (males) or 6 years old (females). These findings indicated that *C*. *longimanus* attend similar isotopic signatures of their mothers at 5–6 years old. The isotopic signature of newborn shark could be used as an indicator of the food sources of their mothers (Elorriaga‐Verplancken et al., 2013).

It should be noted that variance in isotopic compositions from bulk tissue (e.g., vertebra) may reflect mixed effects, such as primary producers, trophic position, and metabolic routing (Magozzi et al., 2021). Compound‐specific stable isotope analysis of amino acids (CSIA‐AA) has been increasingly employed in trophic ecology research (Magozzi et al., 2021; McMahon et al., 2015), since it can reduce uncertainty in estimates of change in location, diet, and nutrient source. Future studies are needed to confirm the results of this study using CSIA‐AA.

### 4.3 Niche width and overlap

The trophic niche width is associated with diversity and availability of food resources which were considered to be the most important factors in shaping trophic niche (Bearhop et al., 2004; Páez‐Rosas et al., 2020). The broad SEA_B_ values suggested diversity of food sources and habitat utilization. The difference exhibited by males and females suggested their complex foraging and migratory behavior. The expansion on isotopic niche of large pelagic sharks due to the opportunistic feeding was reported by Vaudo and Heithaus (2011). Larger niche width of females across growth stages indicated their presence in higher productive areas with abundant resource than the males and possibly move horizontally and vertically within the greater range during growth of the same overall ecosystem. The isotopic niche overlap can reflect the similarity in resource utilization and potential competition among individuals. And the high degree of isotopic overlap between sexes at each growth stages in this study suggested both sexes similar prey species.

## 5 CONCLUSION

The SIA of oceanic whitetip shark (*C*. *longimanus*) vertebrae provided lifetime records of diet and revealed feeding patterns. In this study, similar trends in δ^13^C and δ^15^N values of both sexes were observed, and a high degree of isotopic overlap was found among its entire life histories. Stable isotopic data suggested that *C*. *longimanus* at different stages share similar feeding strategies and both sexes have similar trophic positions in the study area. Moreover, we found that *C*. *longimanus* might occupy different habitats between sexes due to their migratory behaviors. However, it remains unknown whether this sexual dimorphism is prevalent across its geographical range or what environmental conditions trigger sexual segregation in habitat. Further research is needed to understand differences in physiological response and habitat use between males and females.

## CONFLICT OF INTEREST

The authors declare that they have no conflict of interest.

## AUTHOR CONTRIBUTIONS

**Yongfu Shen:** Data curation (equal); Formal analysis (equal); Methodology (equal); Writing – original draft (equal). **Yi Gong:** Supervision (equal); Writing – review & editing (equal). **Feng Wu:** Resources (equal). **Yunkai Li:** Conceptualization (equal); Funding acquisition (equal); Project administration (equal); Supervision (equal); Writing – review & editing (equal).

## Data Availability

Data used in this manuscript were submitted to Dryad and are preliminarily available at https://doi.org/10.5061/dryad.pvmcvdnm6.
